# pH-Dependence Cytotoxicity Evaluation of Artepillin C against Tumor Cells

**DOI:** 10.3390/life13112186

**Published:** 2023-11-09

**Authors:** Wallance M. Pazin, Renata R. Miranda, Karina A. Toledo, Frank Kjeldsen, Carlos J. L. Constantino, Jonathan R. Brewer

**Affiliations:** 1 Department of Physics and Meteorology, School of Sciences, São Paulo State University (UNESP), Bauru 17033-360, Brazil; wallance.pazin@unesp.br; 2 Department of Biochemistry and Molecular Biology, University of Southern Denmark, 5230 Odense, Denmark; renatam.bio@gmail.com (R.R.M.); frankk@bmb.sdu.dk (F.K.); 3 Department of Biological Sciences, School of Sciences, Humanities and Languages, São Paulo State University (UNESP), Assis 19806-900, Brazil; karina.toledo@unesp.br; 4 Department of Physics, School of Sciences and Technology, São Paulo State University (UNESP), Presidente Prudente 19060-900, Brazil; carlos.constantino@unesp.br

**Keywords:** Artepillin C, Brazilian green propolis, antitumor activity, live cell imaging

## Abstract

Brazilian green propolis is a well-known product that is consumed globally. Its major component, Artepillin C, showed potential as an antitumor product. This study explored the impact of Artepillin C on fibroblast and glioblastoma cell lines, used as healthy and very aggressive tumor cell lines, respectively. The focus of the study was to evaluate the pH-dependence of Artepillin C cytotoxicity, since tumor cells are known to have a more acidic extracellular microenvironment compared to healthy cells, and Artepillin C was shown to become more lipophilic at lower pH values. Investigations into the pH-dependency of Artepillin C (6.0–7.4), through viability assays and live cell imaging, revealed compelling insights. At pH 6.0, MTT assays showed the pronounced cytotoxic effects of Artepillin C, yielding a notable reduction in cell viability to less than 12% among glioblastoma cells following a 24 h exposure to 100 µM of Artepillin C. Concurrently, LDH assays indicated significant membrane damage, affecting approximately 50% of the total cells under the same conditions. Our Laurdan GP analysis suggests that Artepillin C induces autophagy, and notably, provokes a lipid membrane packing effect, contributing to cell death. These combined results affirm the selective cytotoxicity of Artepillin C within the acidic tumor microenvironment, emphasizing its potential as an effective antitumor agent. Furthermore, our findings suggest that Artepillin C holds promise for potential applications in the realm of anticancer therapies given its pH-dependence cytotoxicity.

## 1. Introduction

Natural products derived from diverse organisms long held potential as therapeutic agents, and in recent years, the exploration of bioactive compounds from natural sources as potential anticancer agents gained global prominence [1]. With cancer being a leading global cause of mortality, accounting for nearly 10 million fatalities in 2020 according to the World Health Organization, the need for effective treatments is pressing [2]. Natural products offer a promising source for drug discovery, with approximately 25% of the anti-cancer drugs approved between 1981 and 2019 coming originally from natural products [3]. Atanasov et al. [4] highlighted a significant challenge associated with the large-scale production of therapeutic agents from natural products—the need for ample biological material in order to isolate and characterize the bioactive components. However, these limitations can be mitigated through the indirect collection of the bioactive compounds from natural sources via insects [5].

An example of such natural products is propolis, synthesized by bees using bioactive compounds collected from plants to seal their hives [6]. Propolis has a rich history in folk medicine and garnered substantial attention from the scientific community due to its diverse health benefits, including antioxidant, anti-inflammatory, antimicrobial, immunomodulatory, and antitumor properties, attributed to its plant-derived secondary metabolites [7,8,9,10,11]. Notably, the composition of propolis varies depending on the botanical origin, geographical location, and bee species [12,13,14]. Among the diverse types of propolis found in Brazil, Brazilian green propolis, produced by the *Apis mellifera* bee species, holds particular prominence. This is primarily attributed to its high content of Artepillin C (3,5-diprenyl-4-hydroxycinnamic acid), a phenolic acid derivative primarily found in the exudates of *B. dracunculifolia* D.C., commonly known as “alecrim-do-campo”, a native Brazilian plant species prevalent in the southeastern region [15,16]. In a recent review by Shahinozzaman et al. [10], the biological properties of Artepillin C were extensively explored. The review highlighted that this propolis compound is capable of counteracting cellular oxidative mechanisms, serving as a defense mechanism for organisms. Additionally, Artepillin C demonstrated cytotoxicity against cancer cells, revealing its selective efficacy against different cell types.

**Figure 1:**
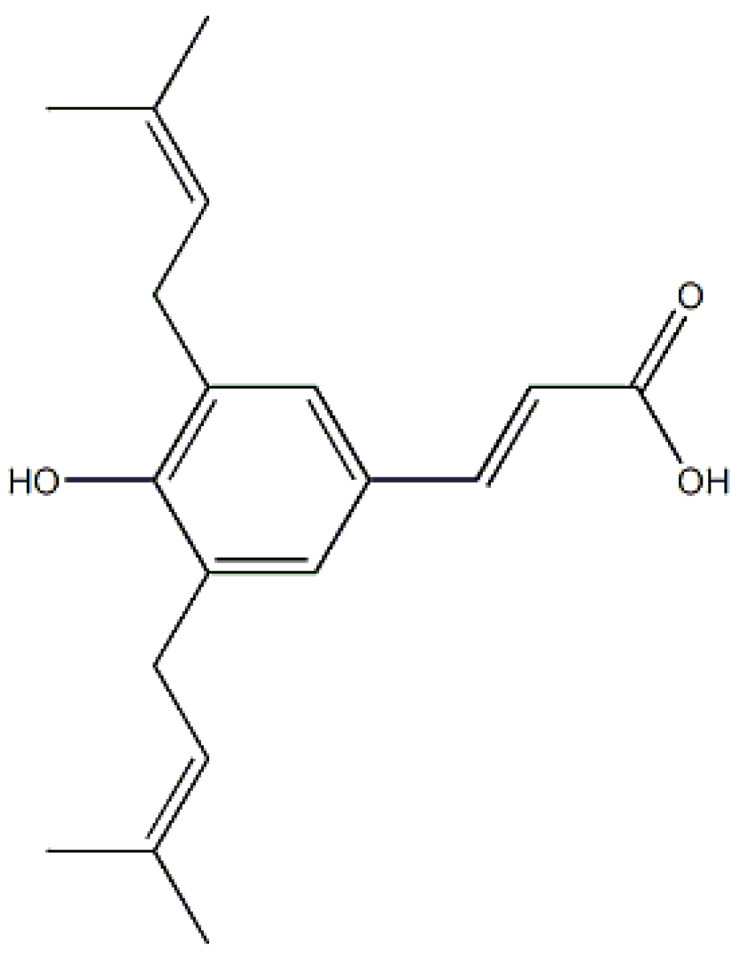
Molecular structure of Artepillin C.

The chemical structure of Artepillin C features two prenylated groups, contributing to its lipophilic nature, as depicted in Figure 1. The core structure primarily comprises a phenolic acid, wherein the carboxyl moiety’s deprotonation is contingent upon the surrounding pH. Notably, the reported pKa value of Artepillin C is 4.65, with its lipophilicity varying in correspondence to its protonation state [17,18]. Within this context, it is pertinent to underscore the acidic attributes of the extracellular microenvironment in tumor cells. This acidity is a consequence of lactate excretion from the cytosol due to the prevalence of the aerobic glycolytic pathway for energy production, a phenomenon known as the Warburg effect [19,20]. Interestingly, this metabolic shift persists regardless of molecular oxygen levels in cancer cells, leading to a substantial elevation in lactate production—up to 40 times higher compared to healthy cells [21]. A previous study showed that models mimicking healthy and tumor cell membranes exhibit distinct responses based on lipid composition and media pH [18,22]. Under physiological conditions (pH 7.4), Artepillin C exhibits an affinity for the lipid assembly without perturbing the structural integrity of the lipid membrane. This suggests a potentially beneficial interaction that could safeguard cell membranes against oxidative stress, as recently reported by our research group [23]. Conversely, exposure to an acidic environment renders Artepillin C hydrophobic, enhancing its interaction with model membranes and subsequently influencing their properties. This observation reinforces the notion that, in addition to the prenylated groups, the protonation state of the carboxyl group contributes to the nuanced biological activity of Artepillin C.

In the current study, we explore the cytotoxic potential of Artepillin C across cell types representing both healthy and tumor lineages, with controlled pH levels in the culture media. Human fibroblasts from healthy subjects were used in this study as representatives of normal tissue, while glioblastoma, a very aggressive malignant brain tumor, was selected to represent the tumor cells under investigation. The assessment of cytotoxicity was conducted through the deployment of the 3-(4,5-dimethylthiazol-2-yl)-2,5-diphenyltetrazolium bromide (MTT) and lactate dehydrogenase (LDH) viability assays. These traditional assays were complemented by live cell imaging utilizing microscopy techniques [24]. To delve deeper into the mechanistic aspects of Artepillin C’s impact on cells, Laurdan GP analysis was integrated to probe alterations in cell membrane packing [25].

It is worth mentioning that glioblastoma represents approximately 57% of all gliomas (accounting for more than 80% of all malignant cerebral neoplasms), and is the most common primary intracranial tumor, considered as grade IV according to the WHO [26]. It is known that 90% of glioblastoma cases are diagnosed as IDH wild type in older patients aged 62 or over, and that the remaining approximately 10% correspond to secondary glioblastoma, that progressively develops from low-grade astrocytoma and frequently manifests in patients aged 40–50 years of age [27]. Current treatments for glioblastoma, ranging from surgeries to radiation therapies and chemotherapy, yielded disheartening prognoses [28,29]. In the context of glioma, it came to light that the pH of the extracellular microenvironment in in vivo cells remains at an acidic level of approximately 5.9 [30]. This finding aroused our interest in modulating the pH of the culture media to a threshold of 6.0. This strategic adjustment enabled us to discern the cytotoxic potential of Artepillin C, aligning with the conditions encountered within the glioma environment.

In summary, the current study describes a comprehensive examination of Brazilian green propolis, a globally recognized natural product, with a primary focus on its major constituent, Artepillin C, which exhibits notable antitumor potential. Through the judicious selection of experimental techniques, including MTT and LDH assays for viability assessment, as well as advanced fluorescence microscopy techniques, such as Laurdan GP, confocal microscopy, and coherent anti-Stokes Raman spectroscopy (CARS), we aimed to provide a rigorous and thorough exploration of the cytotoxic properties of Artepillin C. By investigating its impact on both fibroblast and glioblastoma cell lines, representative of normal and highly aggressive tumor cells, respectively, this research uncovers a nuanced aspect of cytotoxicity, with a specific focus on pH-dependent responses. The outcomes presented in this study serve to validate the affinity of Artepillin C for inducing cytotoxicity within the acidic extracellular microenvironment, which is characteristic of tumor cells. Furthermore, these findings underscore the methodical selection and scientific merit of the chosen experimental techniques in elucidating the therapeutic potential of Artepillin C, thus contributing significantly to the body of knowledge, with implications for potential applications in the realm of anticancer therapies.

## 2. Materials and Methods

### 2.1. Artepillin C Solubilization

Artepillin C (98.0+% HPLC grade—Figure 1) was purchased from Wako (Osaka, Japan) and used without further purification. Stock solution at 50 × 10^−3^ M was made in methanol (analytical grade, Sigma Aldrich, St. Louis, MI, USA) and aliquots were added in culture media to reach the desired concentrations ranging from 1 to 500 µM.

### 2.2. In Vitro Culture of Fibroblasts and Glioblastoma Cells

Fibroblast cells from a human healthy male were purchased from Coriell Institute for Medical Research Biobank (#GM08680; Camden, NJ, USA), while the human glioblastoma cell line U-87 MG was kindly provided by Professor Barbara Guerra (BMB, SDU, Odense, Denmark). Both cell lines were cultured as a monolayer in Dulbecco’s modified Eagle’s medium (DMEM) and 10% fetal bovine serum (FBS), acquired from GIBCO BRL (Life Technologies, Paisley, Scotland), supplemented with 1% penicillin-streptomycin, and kept in a humidified incubator at 37 °C in an atmosphere of 5% CO_2_. For the experiments, cells were plated either in microscope dishes (Ibidi, Munich, Germany) for morphological and LAURDAN GP analyses or 96-well plates for MTT and LDH assays. The pH of the culture media was adjusted to reach 7.4, 7.0, 6.8, 6.5, and 6.0 through the addition of hydrogen chloride (HCl).

### 2.3. MTT and LDH Assays

3-(4,5-dimethylthiazol-2-yl)-2,5-diphenyltetrazolium bromide (MTT) (ref. M2003; Sigma Aldrich) was used to investigate the metabolism stress of fibroblast and glioblastoma cells [31], while cellular membrane damage was evaluated through the release of the enzyme lactate dehydrogenase (LDH) to the culture media, upon Artepillin C and pH treatment [32]. Briefly, cells (1.2 × 10^4^ cells/well) were seeded in a 96-well plate and grown for 24 h. Afterwards, the cells were incubated for 24 h with culture media at the worked pH values and Artepillin C in different concentrations, and diluted in cell culture media (max. 0.5% *v*/*v* MeOH/cell culture media volume to avoid cytotoxicity of the solvent according to previous tests not shown). Cells cultured at normal conditions (established pH at 7.4, without Artepillin C) were considered 100% viable as cellular control (CC).

At the end of the exposure period, the culture medium was replaced by a fresh complete medium containing MTT at 0.5 mg/mL. After incubation for 2 h, cells were washed with PBS and formazan crystal was solubilized with DMSO. The absorbance was measured using a FLUOstar Omega microplate photometer (Ortenberg, Germany) at 570 nm. The LDH assay was performed following the manufacturer’s instructions and fluorescence was measured at 560 nm excitation and 590 nm emission. Cytotoxicity was expressed as the percentage of maximum LDH release. The viability percentage of the cells in different concentrations of Artepillin C was calculated considering three independent biological experiments, with three replicates per experiment. Statistical comparisons between groups were performed using one-way analysis of variance (ANOVA), followed by the Dunnett post hoc test, by means of GraphPad Prism^®^ 5.0 software (GraphPad Software Inc., San Diego, CA, USA). Data are expressed as mean ± standard deviation and *p*-values lower than 0.05 were considered statistically significant.

### 2.4. Microscopy Analyses

Cells (4 × 10^4^ cells/dish), after different incubation time-points (1, 4, 8, 12, and/or 24 h, depending on the analyses), in the presence or absence of 100 µM of Artepillin C, were stained with DiIC_18_ (morphology assays) or Laurdan (Laurdan GP assays). For this, the stock solution of the desired probe (2.5 mg/mL in methanol) was diluted in PBS (1:2000 *v*/*v*), and the final solution was added to the cells. After 5 min of incubation, the probe solution was removed, the cells were rinsed, preserved in PBS, and immediately taken to the microscope to acquire live cell images. For the morphology tests, we used a Leica TSC SP8 STED microscope, equipped with a 40× objective (NA 1.1, water immersion). For DiIC_18_ excitation, a white light laser was tuned at 530 nm. For CARS signal, the microscope was equipped with a picosecond (ps) pulsed optical parametric oscillator (OPO) (Pico Emerald, APE, Berlin, Germany) which delivered a Stokes beam at 1064 nm and tunable pump beam. The pump laser was tuned at 909.3 nm, which combined with the Stokes beam at 1064 nm corresponds to a vibrational wavenumber of 1599 cm^−1^. Light was detected using a CARS detector photomultiplier tube in the epi-direction.

To acquire Laurdan GP images, we used a custom-built multiphoton excitation microscope constructed on an Olympus IX70 microscope [33]. A femtosecond Ti:Sa laser (Broadband Mai Tai XF-W2S with 10W Millennia pump laser, tunable excitation range 710–980 nm, Spectra Physics, Mountain View, CA, USA) tuned at 780 nm was used as an excitation wavelength source. The objective used in the experiments was a 60× water immersion, NA of 1.2. The fluorescence signal was collected in two separate detectors (Hamamatsu H7422P-40) by splitting the fluorescence with a dichroic mirror above and below 475 nm. Each detector contained additional bandpass filters (438 ± 12 nm and 494 ± 10 nm) allowing simultaneously acquisition of intensity images corresponding to the blue and red sides of the LAURDAN spectrum (i.e., *I*_440_ and *I*_490_, respectively), which are necessary to compute the GP images according to the equation:

(1) $$GP=\frac{I_{440}-I_{490}}{I_{440}+I_{490}}$$

where *I*_440_ and *I*_490_ correspond to the fluorescence emission of the LAURDAN at 440 and 490 nm, respectively [26]. The calculated GP images were calibrated with a correcting factor G since GP values, obtained from the GP images, strongly depend on instrumental factors, such as filter settings and gain of the PMTs used in the microscope. Therefore, the absolute measurement of the GP was calculated by the calibration of the relative intensity of the channels. The equation utilized to calculate the GP images, considering the correcting factor G is:

(2) $$GP=\frac{I_{440}-(G\times I_{490})}{I_{440}+{(G\times I}_{490})}.$$

The GP from Equation (1) is then corrected using the G-factor value acquired from the images of the LAURDAN reference solution in DMSO using the same instrumental conditions as in the live cell experiments. This value must match the value calculated from the absolute GP measured using a fluorometer. The G-factor value can be calculated from the following equation:

(3) $$G=\frac{I_{440}(1-GPc)}{I_{490}(GPc+1)}$$

where GPc is the GP value from the reference solution measured in the fluorometer (ISS, Champaign, IL, USA) at the emission wavelengths defined for Equation (1). The excitation wavelength was 374 nm and the emission wavelengths 440 and 490 nm.

## 3. Results and Discussion

### 3.1. Cell Viability Studies

**Figure 2:**
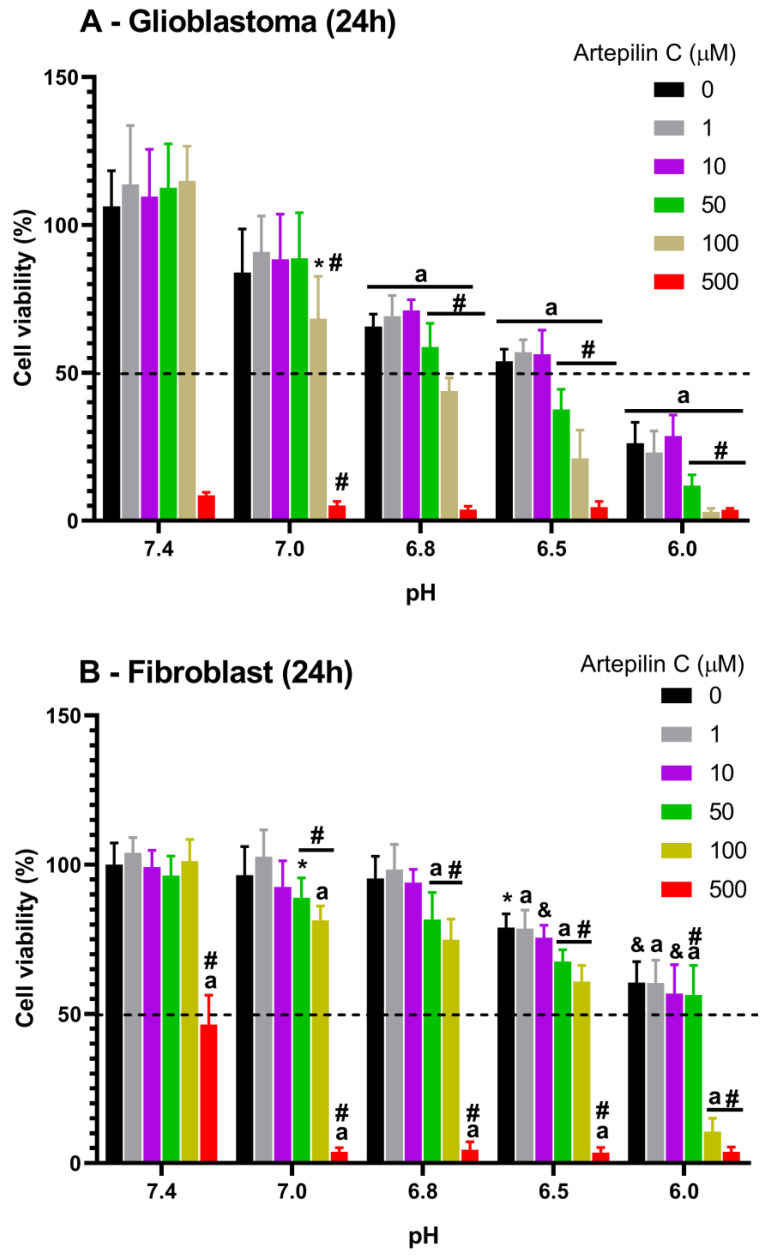
MTT-based cell viability assay of glioblastoma (**A**) and fibroblast (**B**) cells at different pH values after 24 h incubation with 0, 1, 10, 50, 100, and 500 µM of Artepillin C. * for *p* < 0.05, & for *p* < 0.01, a for *p* < 0.001 compared to the general control (0 µM Artepillin C, pH 7.4); *#* denotes significant difference between the groups and their specific pH control (0 mol/L Artepillin C, respective pH value).

The MTT assay was initially employed to evaluate the impact of Artepillin C on glioblastoma tumor cells under diverse concentration and pH conditions. The incubation period spanned 24 h, as illustrated in Figure 2A. At pH 7.4, Artepillin C does not influence cellular metabolic activity up to 100 µM, in contrast to the highest studied concentration, 500 µM, which elicited a striking inhibition exceeding 90% of cell viability, regardless of the culture media pH. At pH 7.0, the absence of Artepillin C (0 µM) led to a marginal 15% reduction in cellular viability compared to pH 7.4, although not statistically different. This pattern persisted for assays conducted in the presence of Artepillin C at the same pH level, relative to pH 7.4, for concentrations as high as 50 µM. However, at 100 µM of Artepillin C, cellular viability diminished to 68% ± 14%, underscoring its cytotoxic activity at pH levels marginally lower than 7.4.

Considering the aforementioned, it becomes evident that pH significantly influences the metabolic activity of cells, manifesting as a series of descending bars representing measurements in the absence of Artepillin C from pH 6.8 downward. Thisdecrement culminates in values below 50% at pH 6.0 (illustrated by the black bar denoting pH 6.0 in Figure 2A). Furthermore, for Artepillin C concentrations of 1 and 10 µM incubated with cells at pH 6.0, viability remained statistically indistinguishable from that observed in the absence of Artepillin C at the corresponding pH. This suggests that, for these concentrations, Artepillin C does not exert cytotoxic effects even within the most acidic milieu explored. The literature supports the notion that Artepillin C favors a neutral state as pH decreases [17]. Beyond 50 µM, molecules tend to aggregate due to their limited water solubility, augmenting interactions with lipophilic domains, such as the cell plasma membrane, and subsequently intensifying cytotoxic effects [18,22,34]. Notably, the decline in cellular viability observed at pH values of 6.8, 6.5, and 6.0 when cells were exposed to Artepillin C concentrations exceeding 50 µM, marked by significant differentiation from the respective pH controls, clearly underscores the pH-dependent effect. A noticeable trend emerges: a lower pH corresponds to heightened Artepillin C-induced cytotoxicity. This phenomenon is even more pronounced with escalated concentrations—viability receded to approximately 68% at pH 6.8, to 40% at pH 6.5, and a mere 13% at pH 6.0 (relative to the control 0 µM Artepillin C for each pH scenario). 

Simultaneously, a parallel analysis was conducted on fibroblasts, chosen as a representative healthy cell culture control for comparison with the effect of Artepillin C observed in glioblastomas, as depicted in Figure 2B. A congruent pattern to that observed in glioblastoma cells emerged when fibroblasts were exposed to Artepillin C at pH 7.4, with one exception: after incubation with 500 μM of Artepillin C, a reduction in cell viability of approximately 50% was noted, suggesting resistance of healthy cells to elevated concentrations of Artepillin C in physiological conditions. This intriguing finding implies that, in low concentrations, Artepillin C does not exhibit cytotoxicity in healthy cells, thereby supporting its potential as an antioxidant product derived from Brazilian green propolis [23]. 

The pH-dependent effect on cell viability in the absence of Artepillin C was specifically pronounced at the two lowest pH values in this study, i.e., 6.5 and 6.0. This influence persisted even when cells were incubated with 1 or 10 µM of Artepillin C. However, in concentrations exceeding 50 µM, a distinct effect was observed. At pH 7.0, some degree of cytotoxicity manifested, intensifying notably at pH 6.0, especially in cases where Artepillin C concentrations exceeded 100 µM. Studies exploring pH-responsive cytotoxicity against tumor cells were reported considering new strategies for future clinical trials, given the importance of pH in the cancer microenvironment [35,36,37,38,39,40].

The data derived from the MTT assays clearly underscore the interplay between pH and the cytotoxicity potential of Artepillin C. Under physiological conditions (pH 7.4), Artepillin C exists in its monomeric form [17], with cytotoxicity becoming evident only at the highest studied concentration, regardless of whether applied to glioblastoma or fibroblast culture. Intriguingly, healthy cells exhibit greater resistance to the high concentration of Artepillin C at pH 7.4 when juxtaposed with tumor cells. Furthermore, analysis of cell viability in the absence of Artepillin C (0 µM) reveals that decreasing pH values correspond to an increase in cytotoxicity, which is particularly discernible in glioblastoma cultures. 

Notably, at pH 6.0, the viability of fibroblasts in the absence of Artepillin C (Figure 2A—black bars) diminished to 24% ± 8% relative to the negative control (pH 7.4—absence of Artepillin C). Strikingly, when exposed to 100 µM of Artepillin C at the same pH of 6.0, viability diminished to 3% ± 1%, marking an approximate 8-fold reduction compared to values observed without Artepillin C at this pH. To more accurately represent this trend, a statistical analysis normalizing the viability determined at pH 6.0 in the absence of Artepillin C (considered as 100%) was undertaken. This normalization was related to cell cultures incubated with the varying concentrations of Artepillin C (see Supplementary Figure S1). Notably, for both 100 and 500 µM concentrations of Artepillin C, a substantial reduction in viability was noted across both fibroblast and glioblastoma cell lines in relation to the control (no Artepillin C) at pH 6.0 (approximately 25% and 17% for the fibroblast cell line at 100 and 500 µM, respectively; and below 12% for both concentrations in the case of glioblastoma). Impressively, even at 50 µM, glioblastoma cell viability exhibited a considerable reduction (approx. 58%), further affirming the pH-dependent cytotoxic influence of Artepillin C on cells, particularly within the glioblastoma cell line.

**Figure 3:**
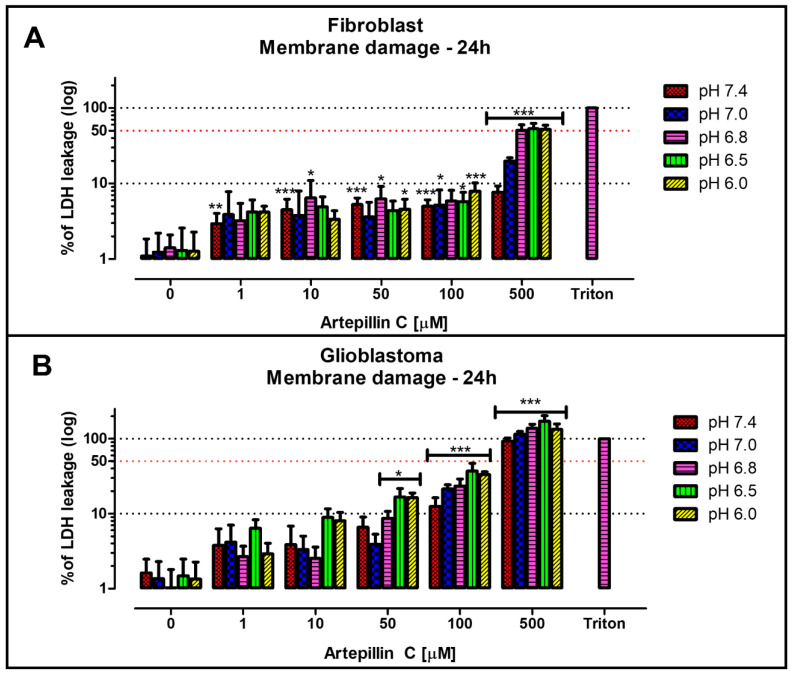
LDH assay representing the percentage of membrane integrity damage obtained for fibroblast (**A**) and glioblastoma (**B**) cell lines, after 24 h incubation, exposed to 0, 1, 10, 50, 100, and 500 µM of Artepillin C. Triton was used as the positive control for membrane damage assays, since it solubilizes lipids, releasing 100% of LDH. * for *p* < 0.05, ** *p* < 0.01 and *** for *p* < 0.001 compared to the general control (0 µM Artepillin C, pH 7.4).

To ascertain whether the actions of Artepillin C correlate with plasma membrane damage, LDH leakage analyses were conducted. This was particularly pertinent since literature reports indicated an increase in the lipophilicity of Artepillin C in acidic environments [34]. As a positive control (100% LDH leakage), Triton X-100, a surfactant and membrane permeabilization agent [41], was introduced. Insights gleaned from these data reveal that the observed effects of acidic cell culture media on cellular viability assays, in the absence of Artepillin C, do not induce membrane impairment. Across all pH values studied for both fibroblast and glioblastoma cultures (as depicted in Figure 3A and Figure 3B, respectively), the LDH leakage remained under 2%. Notably, in fibroblasts, the maximum percentage of leakage did not surpass 10% up to 100 µM of Artepillin C for all tested pH media. In contrast, the glioblastoma cell line exhibited leakage values reaching up to 50% for 100 µM of Artepillin C at lower pH levels. Interestingly, after incubation with 500 µM of Artepillin C, glioblastoma cells exhibited LDH leakage approaching 100% across all examined pH values. This suggests that the profound cytotoxicity observed in cellular viability assays stems from disruption of the membrane integrity. 

In the context of fibroblasts at pH 7.4, while membrane integrity was indeed compromised by incubating cells with 500 µM of Artepillin C (as seen in Figure 3A), the most pronounced effects were associated with mechanisms related to metabolic activity, mirroring the outcomes of the MTT assays. Remarkably, at pH 7.4, a loss of 50% cellular viability was recorded for the highest concentration of Artepillin C tested in healthy cells (500 µM). Figure 3A indicates that, under identical conditions, a mere 10% LDH leakage was discerned. At lower pH values, metabolic activity experienced complete cessation (as indicated in Figure 2). Conversely, a 50% LDH leakage was observed (as shown in Figure 3A). This hints at the possibility that disparities in membrane lipid composition between healthy and tumor cells, even within acidic media, may play a pivotal role in the cytotoxicity exerted by Artepillin C. 

Insights from a prior study further accentuate this point: the effects of lipid membrane permeability in giant unilamellar vesicles (GUVs) were more pronounced when anionic lipid phosphatidylserine (PS) was incorporated in the composition, as opposed to a pure zwitterionic PC composition. Specifically, the inclusion of 20% DPPS in the DPPC GUVs composition expedited leakage from the vesicles after introducing 100 µM of the neutral structure of Artepillin C. This suggests the involvement of anionic lipids in Artepillin C’s interaction with the lipophilic milieu [34]. 

### 3.2. Live Cell Imaging

To explore the interplay between pH and Artepillin C in cell morphology, a comprehensive examination was conducted through live cell imaging. This investigation encompassed both fibroblast and glioblastoma cell lines, cultivated in culture media featuring pH extremes of 7.4 and 6.0, which were carefully selected to comprise the boundaries of the aforementioned biological assays. To ensure a consistent comparative foundation, the concentration of Artepillin C was uniformly set at 100 µM. This decision was supported by the substantial disparities unveiled by the results of cellular viability and membrane integrity assays, observed across both fibroblast and glioblastoma cell lines, under both pH conditions, in the presence and absence of Artepillin C at this specific concentration.

**Figure 4:**
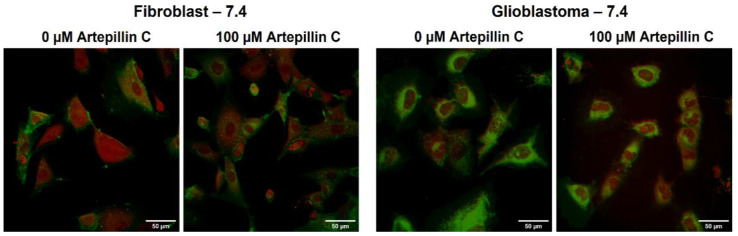
Confocal fluorescence (green) and CARS microscopy (red) images of fibroblast and glioblastoma living cells collected after 24 h of incubation, at pH 7.4, in the absence and presence of 100 µM of Artepillin C. The images are representative of samples analyzed in triplicate.

Figure 4 showcases a collection of illustrative images derived from the live cell imaging of fibroblast and glioblastoma cells, captured after a 24 h incubation period at pH 7.4. These images were acquired through two distinct microscopy techniques: confocal fluorescence (highlighting the plasmatic membrane through DiIC_18_ labeling with excitation at 530 nm) and CARS microscopy. The latter technique involved excitation at 1599 cm^−1^, a wavenumber corresponding to the vibrational motion of proteins [42,43], which enabled us to discern cellular morphology. From both microscopy methodologies, a notable observation emerges. At pH 7.4, a remarkable similarity in cell shapes prevails, irrespective of the absence or presence of 100 µM of Artepillin C. This concordance underscores the congruence of the cellular morphological patterns with the non-cytotoxic effects established via the MTT assays, which were conducted under analogous conditions. 

**Figure 5:**
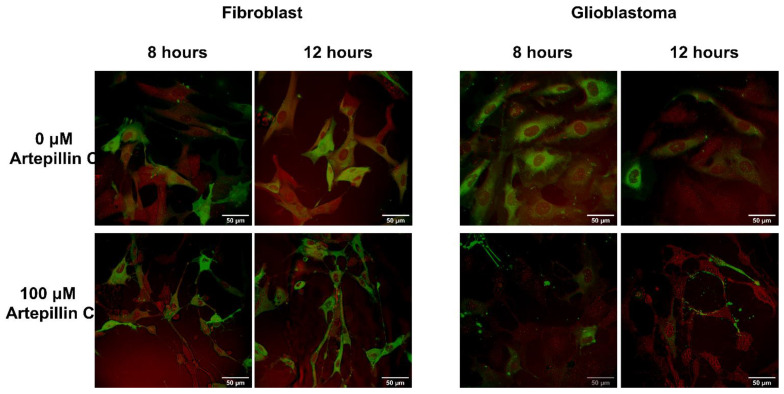
Confocal fluorescence (green) and CARS microscopy (red) images of fibroblast (**left**) and glioblastoma (**right**) images were collected after 8 h and 12 h of Artepillin C incubation, at pH 6.0, in the absence and presence of 100 µM of Artepillin C. The images are representative of samples analyzed in triplicate.

To capture early stage morphological alterations preceding the maximum loss of cell viability observed at the 24 h mark under pH 6.0 conditions, a distinct set of experiments was conducted. Live cell imaging was initiated at 1, 4, 8, and 12 h intervals. In order to provide a comprehensive picture, a comparison was drawn between the negative control and cells subjected to incubation with 100 µM of Artepillin C for durations of 8 and 12 h (Figure 5). Cell images at 1 and 4 h are available in Supplementary Figure S2.

Noteworthy disparities emerge for both the fibroblast and glioblastoma cell lines across the designated incubation periods. Notably, control cells incubated without Artepillin C exhibit a morphological structure akin to that observed at pH 7.4, implying that, over a span of up to 12 h, pH-induced effects on cells remain imperceptible. However, after incubation with 100 µM of Artepillin C, discernible transformations become evident in both cell types.

Intriguingly, the cellular form becomes notably elongated, accompanied by the emergence of filopodia (elongated membrane protrusions), a distinctive manifestation attributable to the influence of Artepillin C, as is readily apparent in Figure 5. This morphological shift assumes heightened prominence in glioblastomas, where membrane staining becomes arduous, and the underlying structural arrangement of proteins undergoes disruption, as evidenced by the outcomes of CARS microscopy.

The findings collectively point to the influence of Artepillin C on the plasma membrane of cells. Consequently, to glean insights into the membrane fluidity of these cells, Laurdan GP imaging experiments were undertaken from live fibroblasts and glioblastoma cells incubated across varied time spans at pH values of 7.4 and 6.0. It is important to highlight that Laurdan, at low concentrations, as used in this study, is appropriate to acquire live cell imaging, providing insights into the membrane order by measuring LAURDAN partitioning and its dynamics [44].

**Figure 6:**
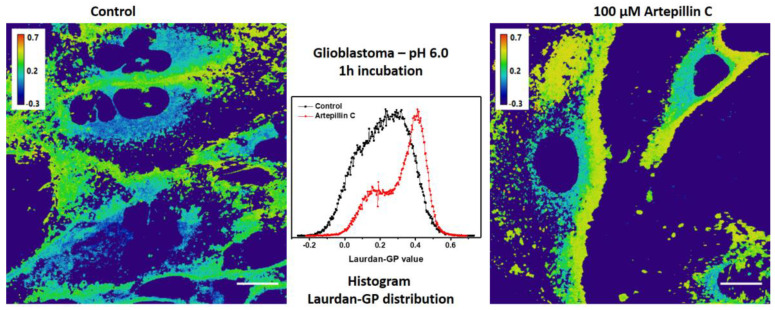
Laurdan GP images and histogram obtained for glioblastoma incubated for 1 h at pH 6.0, in the absence (control—**left**) and presence (**right**) of 100 µM of Artepillin C. Scale bars 50 µm.

Calculations were rooted in the histogram of pixel distribution across a range of Laurdan GP values for each image, as showcased in Figure 6, for a 1 h exposure to Artepillin C. Intriguingly, when subjected to a pH of 7.4 and exposed to 100 µM of Artepillin C, fibroblast Laurdan GP values exhibited augmentation in comparison to values computed from images of control cells devoid of Artepillin C. It is crucial to acknowledge that Laurdan permeates both cytoplasmic membranes and organelles within the cytosol. As a result, the GP distribution is inherently linked to contributions from both of these regions. The higher value of Laurdan GP in glioblastoma (0.23 ± 0.01) compared to fibroblast cells (0.14 ± 0.02) in normal conditions (pH 7.4, in the absence of Artepillin C) seems to be related to the higher amount of cholesterol esters and triacylglycerols compared to non-cancer cells [45].

By meticulously defining the cell membrane as the exclusive region of interest within the image, it becomes evident that, at pH 7.4, the Laurdan GP value remains unaltered for both healthy and tumor cells, irrespective of the presence of Artepillin C. This outcome underscores that, under these conditions, the lipid packing of the membrane experiences no noteworthy alteration in response to Artepillin C (Table 1, enclosed values within each box for respective conditions). These insights substantiate the observations gleaned from the MTT and LDH assays, alongside the cellular morphology, thereby accentuating the non-cytotoxic nature of Artepillin C at this specific concentration and condition of 100 µM.

The discernible elevation in the mean GP value, as evidenced by the histogram depicted in Table 1 following a 24 h incubation of Artepillin C at pH 7.4, is directly attributed to Laurdan residing within the cytosol. This phenomenon arises due to Artepillin C inducing autophagy within the cells, a process that leads to the progressive transportation of Artepillin C to the lysosomes over the course of incubation. Consequently, this contributes to the heightened Laurdan GP value [46]. 

Considering Laurdan GP values computed for both fibroblast and glioblastoma cells at pH 6.0 across intervals of 1, 4, and 8 h, a salient trend emerges. These values are significantly elevated in the presence of Artepillin C when compared to the control cells, as highlighted in Table 1 (values located at the top of each box). Beyond the instigation of autophagy, as elucidated for pH 7.4, the augmentation in GP values specifically within the cell membrane region (as illustrated in Table 1, values at the bottom of each box) serves as a clear indicator of intensified lipid packing due to Artepillin C, persisting across all observed incubation durations.

Notably, within the region of cell membranes, despite the increased GP value, this parameter remains unchanged, independent of the span of incubation. However, in the context of the analysis focusing on values calculated exclusively within the cytosol, a substantial increase is documented over time. This increase peaks at the 8 h mark of Artepillin C incubation, a stage highlighted by pronounced lysosome density, as showcased in Supplementary Figure S3. This alignment underscores the prominence of Artepillin C in instigating autophagy induction.

Of particular intrigue are the insights derived from the confocal images, which unequivocally denote that cellular morphology largely remains unaltered up until the 8 h threshold of Artepillin C incubation. However, a significant increase in Laurdan GP values, relative to control cells, is already perceptible after just 1 h. This feature illustrates that while cellular viability persists, there is a cumulative influence of Artepillin C in changing the lipid packing of both cell plasma membranes and internal lipid bodies.

It is important to underscore that the progressive elevation in GP values throughout the course of incubation is indicative of a composite impact stemming from the cell death or stress, attributed to the interplay of both pH and Artepillin C. This aligns with the discussion pertaining to MTT assays, which emphasize the observed cytotoxicity within the cells in the absence of Artepillin C under a pH of 6.0, regardless of the cell line under consideration.

For the time point demarcated at 12 h, intriguing dynamics come to light. In this instance, GP values pertaining to control cells continue to rise, whereas the values corresponding to treated cells experience a decline relative to the values computed for briefer incubation intervals. This collection of data implies that the prevailing pH continues to exert its influence on the untreated cells, while the insights gathered from the treated cells reflect the consequences of cellular demise on GP values. This rationale is reinforced by the outcomes derived from morphological structural analyses, as depicted within confocal images, as well as the insights harnessed from the previously discussed LDH assays.

The results demonstrate the multifaceted interplay between pH, plasma membrane interactions, autophagy induction, and cellular demise. The MTT assays conducted in this study revealed a clear pH-dependent cytotoxicity of Artepillin C, particularly in glioblastoma tumor cells. At concentrations above 50 µM, Artepillin C exhibited a pronounced decrease in cellular viability, with pH levels below 6.8 yielding viability values below 50%. The propensity for Artepillin C to aggregate in lower pH conditions, resulting in intensified interactions with lipophilic domains such as the cell plasma membrane, aligns with the observed cytotoxic effects provided by the increase in LDH leakage in glioblastoma cells at lower pH levels and higher concentrations of Artepillin C, strongly suggesting that the observed mechanism of action arises from the affinity for a lipid environment with disruption of membrane integrity. Cell live imaging confirms the morphological alterations in the cells, at the same pH and Artepillin C condition, including cell elongation and the emergence of filopodia in the membrane of cells. The morphological alterations are accompanied by an increase in Laurdan GP values, indicative of the increase in the lipid packing due to Artepillin C. The progressive elevation in GP values throughout the incubation period underscores the cumulative influence of Artepillin C on cell death or stress, inducing autophagy, leading the cells to death. Therefore, the current study provides a comprehensive exploration of the cytotoxic mechanism of Artepillin C, with a particular emphasis on its pH-dependent response, given that at pH 7.4, none of the discussed effects were pronounced when the cells were exposed to Artepillin C.

## 4. Conclusions

This study delved into the intricate interplay between Artepillin C, cellular membranes, and pH conditions, unraveling compelling insights into its effects on both healthy fibroblast and malignant glioblastoma cell lines. The results underscore the potent influence of Artepillin C on cell viability, elucidating a pH-dependent cytotoxicity, mainly in glioblastoma cells. The pivotal role of cellular membranes was explored through meticulous analyses of membrane integrity, fluidity, and morphological alterations, revealing the dynamic interactions of Artepillin C. Notably, our study revealed the ability of Artepillin C to induce autophagy, prompting a cascade of events, leading to altered membrane fluidity and potential reorganization. The convergence of these findings underscores the multifaceted nature of the Artepillin C action, mediated through a combination of pH effects and inherent molecular properties. The unveiling of the intriguing mechanisms of Artepillin C and the potential implications for future therapeutic strategies stands as a significant step in the cancer research field. Our exploration not only enhances our understanding of this unique natural compound, but also contributes to the broader landscape of molecular biology and biophysics. As the pursuit of novel anticancer agents continues, the intricate relationship between natural compounds, cellular microenvironments, and intricate molecular processes presents an expansive path for further inquiry. The complex interplay involving Artepillin C, pH conditions, and cell membranes prompts future investigations to uncover deeper insights, guiding the pursuit of inventive therapeutic strategies.

## Figures and Tables

**Table 1:** Mean Laurdan GP values obtained from images of fibroblast and glioblastoma living cells at pH 7.4 (in the absence and presence of 100 µM Artepillin C after 24 h of incubation) and pH 6.0 (in the absence and presence of 100 µM Artepillin C after 1, 4, 8, and 12 h of incubation). The average Laurdan GP values were calculated from the mean values of the histograms obtained from the data analyzed regarding three independent measurements, totaling more than 16 evaluated cells. The table presents the GP values calculated for each condition (pH, cell type, and incubation time), taking into account the Laurdan distribution in the whole cell (values at the top of each box) and the Laurdan specifically in the region of the cell membrane (values at the bottom of each box).

pH	Incubation Time	Laurdan GP Values			
		Fibroblasts		Glioblastomas	
		Control	100 µM Artepillin C	Control	100 µM Artepillin C
7.4	24 h	0.14 ± 0.02	0.21 ± 0.03	0.23 ± 0.01	0.26 ± 0.01
		0.35 ± 0.04	0.37 ± 0.05	0.37 ± 0.03	0.35 ± 0.03
6.0	1 h	0.16 ± 0.01	0.26 ± 0.05	0.16 ± 0.01	0.31 ± 0.01
		0.34 ± 0.02	0.37 ± 0.03	0.37 ± 0.01	0.40 ± 0.01
	4 h	0.18 ± 0.01	0.30 ± 0.01	0.19 ± 0.07	0.30 ± 0.01
		0.34 ± 0.01	0.38 ± 0.02	0.37 ± 0.02	0.40 ± 0.01
	8 h	0.21 ± 0.03	0.37 ± 0.02	0.19 ± 0.02	0.35 ± 0.01
		0.33 ± 0.03	0.40 ± 0.01	0.34 ± 0.04	0.40 ± 0.02
	12 h	0.27 ± 0.04	0.27 ± 0.02	0.22 ± 0.04	0.27 ± 0.02
		0.34 ± 0.02	0.31 ± 0.01	0.38 ± 0.01	0.39 ± 0.01

## Data Availability

The data presented in this study are available in this article and supplementary material.

## Supplementary Materials

The following supporting information can be downloaded at: https://www.mdpi.com/article/10.3390/life13112186/s1, Figure S1: Cell viability percentage for fibroblast and glioblastoma cell line at pH 6.0, obtained by means of MTT assay, after 24 h incubation, exposed to 0, 1, 10, 50, 100, and 500 µM of Artepillin C. ** for *p* < 0.05, *** for *p* < 0.001 compared to the relative control (0 µM Artepillin C, pH 6.0); Figure S2: Confocal fluorescence (green) and CARS microscopy (red) images of fibroblast (left) and glioblastoma (right) images were collected after 1 h and 4 h of Artepillin C incubation, at pH 6.0, in the absence and presence of 100 µM of Artepillin C. The images are representative of samples analyzed in triplicate; Figure S3: Laurdan GP image obtained for fibroblast cells incubated for 8 h at pH 6.0, in the presence of 100 µM of Artepillin C. The white and red arrows (lower left) indicate lysosome and cell membrane, respectively. Scale bar 50 µm.
