# Supplementary material for: pH-Dependence Cytotoxicity Evaluation of Artepillin C against Tumor Cells
Source: Life (Basel). 2023 Nov 9;13(11):2186. doi: 10.3390/life13112186 (PMC10672498; doi:10.3390/life13112186)
Supplement: Supplementary file 1 [file life-13-02186-s001.zip › life-2638628-supplementary.pdf]

The pH-dependence on the cytotoxicity of Artepillin C against tumor cells

SUPPORTING INFORMATION

Wallance Moreira Pazin[1,2], Renata R. Miranda[2], Karina A. Toledo[3], Frank Kjeldsen[2], Carlos J. L. Constantino[4] and Jonathan R. Brewer[2,*]

[1] Department of Physics and Meteorology, School of Sciences, São Paulo State University (UNESP), Bauru, SP, Brazil; wallance.pazin@unesp.br (W.M.P)
[2] Department of Biochemistry and Molecular Biology, University of Southern Denmark, Odense, Denmark; renatam.bio@gmail.com (R.R.M); frankk@bmb.sdu.dk (F.K.)
[3] Department of Biological Sciences, School of Sciences, Humanities and Languages, São Paulo State University (UNESP), Assis, SP, Brazil (karina.toledo@unesp.br)
[4] Department of Physics, School of Sciences and Technology, São Paulo State University (UNESP), Presidente Prudente, SP, Brazil; carlos.constantino@unesp.br (C.J.L.C)

* Correspondence: brewer@bmb.sdu.dk; Tel.: ((+45)6550-3505; Syddansk Universitet, Campusvej, 55, 5230, Odense M, Denmark)

Supporting information 1

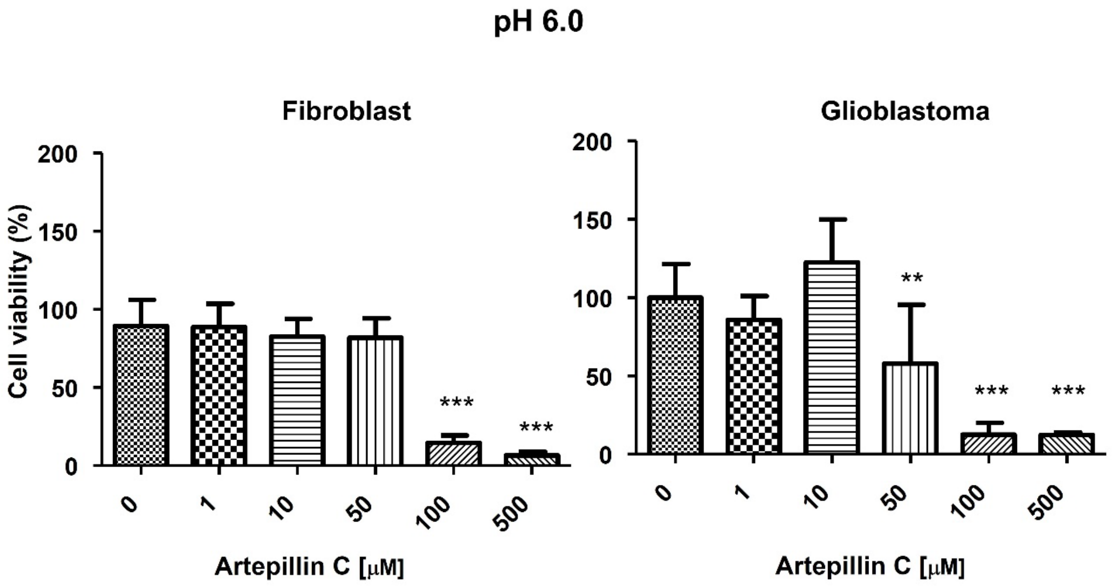

Figure S1. Cell viability percentage for fibroblast and glioblastoma cell line at pH 6.0, obtained by means of MTT assay, after 24h incubation, exposed to 0, 1, 10, 50, 100 and 500 μM of Artepillin C. ** for $p<0.05$, *** for $p<0.001$ compared to the relative control (0 μM Artepillin C, pH 6.0).

Supporting Information 2

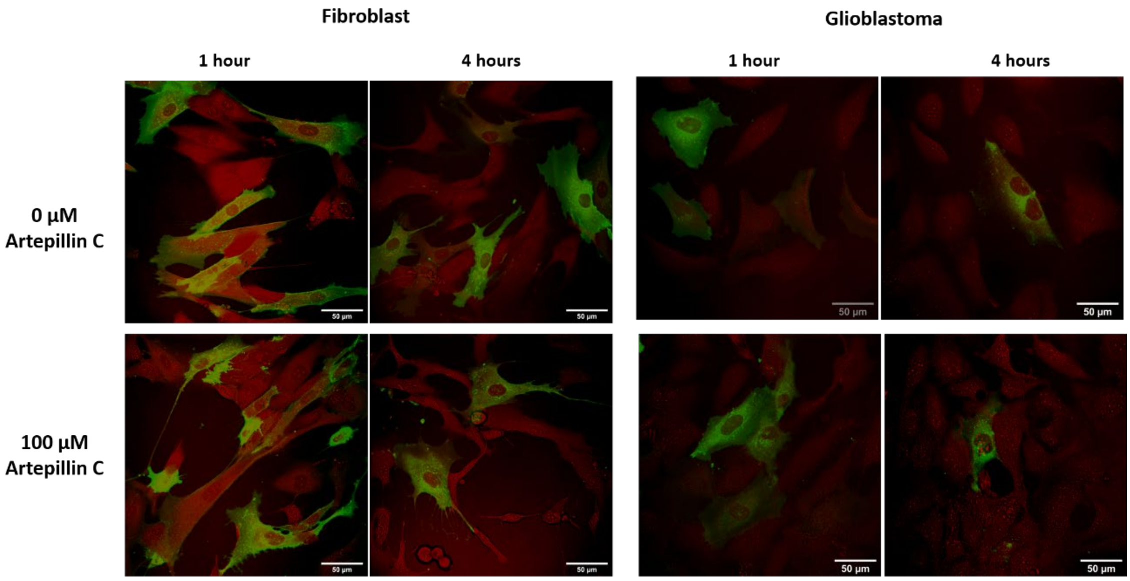

Figure S2. Confocal fluorescence (green) and CARS microscopy (red) images of fibroblast (left) and glioblastoma (right) images were collected after 1h and 4h of Artepillin C incubation, at pH 6.0, in the absence and presence of 100 µM of Artepillin C. The images are representative from samples analyzed in triplicate.

Supporting Information 3

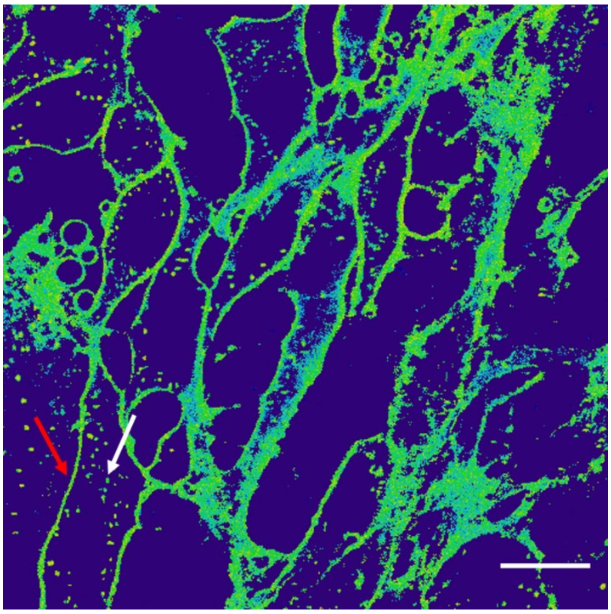

Figure S3. Laurdan-GP image obtained for fibroblast cells incubated for 8h at pH 6.0, in the presence of 100 μM of Artepillin C. The white and red arrows (lower left) indicate lysosome and cell membrane, respectively. Scale bar 50 μm.
